# The relationship between urban and rural health insurance and the self-rated health of migrant workers in Southwest China

**DOI:** 10.1186/s12913-021-06646-3

**Published:** 2021-06-29

**Authors:** Dingying Fu, Li Liu, Xuewen Zhang, Chuan Yu, Huiqiang Luo, Ningxiu Li

**Affiliations:** 1 West China School of Public Health and West China Fourth Hospital, Sichuan University, Chengdu, China; 2 Sichuan Academy of Social Sciences, Chengdu, China; 3 School of Integrated Traditional Chinese and Western Medicine, Jining Medical University, Jining, China

**Keywords:** Self-rated health, Urban and rural health insurance, Migrant workers, China

## Abstract

**Background:**

Following health insurance reforms, China’s health care system has made great progress. However, there are still huge differences between the urban and rural health insurance systems. For rural-to-urban migrant workers, there may be differences in the use of urban and rural health insurance to improve their health status. This study aimed to determine whether any disparities exist in the relationship between urban and rural health insurance and the self-rated health (SRH) of migrant workers in Southwest China from the perspective of urban and rural segmentation.

**Methods:**

Using cross-sectional survey data on Southwest China in 2016, a representative data sample drawn from 8507 migrant workers was analysed. An ordinary least squares (OLS) model and instrumental variable (IV) estimation were used to analyse the relationship between urban and rural health insurance and the SRH of migrant workers.

**Results:**

Using the IV method to control the endogeneity problems associated with health insurance, this study found that there are differences in the relationship between urban and rural health insurance and the SRH of migrant workers. Urban health insurance is associated with significant improvements in the SRH of migrant workers. Compared with the NRCMS, participating in urban health insurance, including urban employee basic medical insurance (UEBMI) and urban resident basic medical insurance (URBMI), increases the likelihood of migrant workers having better SRH.

**Conclusions:**

There are disparities in the relationship between urban and rural health insurance and the SRH of migrant workers in China. Compared to rural health insurance, urban health insurance has a more positive correlation with the health of migrant workers. Our study shows that it is necessary to integrate urban and rural health insurance to promote social equity.

**Supplementary Information:**

The online version contains supplementary material available at 10.1186/s12913-021-06646-3.

## Background

Over the past few decades, with China’s economic reform, China’s economy has grown significantly, and people’s health has improved. However, there are still huge differences in the urban and rural economic development levels. In 2016, the per capita disposable income of urban and rural citizens was 33,616 yuan and 12,363 yuan, respectively [1]. At the same time, previous studies have indicated significant urban-rural inequalities in health-related issues such as health care resources [2, 3] and health outcomes in adults and children [3–5]. Although many factors account for the large gap between the urban and rural environments, access to the health care system is a pivotal factor.

Health insurance, which is based on risk sharing [6], is an essential instrument that can be used to increase the use of medical services and improve population health [7]. To ensure equal access to medical resources and to promote population health, China has implemented a series of health care reforms to establish a universal health insurance system. This system consists of three predominant health insurance schemes, the major distinguishing features of which are that the availability of a specific health insurance plan depends on one’s employment status, household registration (hukou) (urban vs. rural), and place of residence [8]: urban employee basic medical insurance (UEBMI) is for urban formal sector employees, the new rural cooperative medical system (NRCMS) is for rural people, and urban resident basic medical insurance (URBMI) is mainly for urban informal sector workers and unemployed urban residents. Rural health insurance differs from urban health insurance in terms of eligibility, administration, pooling levels and benefit packages (Table 1). UEBMI and URBMI are administered by the Chinese Ministry of Human Resources and Social Security, while the NRCMS is administered by the Chinese National Health Commission. Both UEBMI and URBMI funds are managed at the municipal level, while NRCMS funds are mainly managed at the county level. Compared with urban health insurance, rural health insurance has inferior health care coverage [11] and a lower reimbursement rate. The reimbursement rate of the NRCMS is 10% lower than that of UEBMI and URBMI [10], which is mainly due to the low funds of the NRCMS [12]. This leads to a tiered system in which people who participate in urban health insurance can enjoy access to a wide range of high-quality services, while those who participate in rural health insurance have access to a less generous benefit package and incur higher costs due to co-payments or excluded services [13, 14]. In 2008, the average hospitalization expenses of NRCMS enrolees accounted for 56% of per capita annual income, while the proportions for UEBMI and URBMI enrolees were only 31.8 and 38.2%, respectively [15, 16]. China’s health insurance system, which is characterized by an “urban-rural duality” [17], is a tiered health system, and this system is inequitable because it cannot promote financial protection or an equitable utilization of health services [14, 18]. At the same time, this kind of health insurance system featuring an “urban-rural duality” has also resulted in potential barriers to obtaining appropriate and cost-effective health care for a large number of rural-to-urban migrant workers, who are caught in the gap between urban and rural areas [11, 15]. This situation leads to increasing concerns over whether this unique group has benefited equally from insurance coverage.

**Table 1:** Basic information about the three health insurance schemes in China

	UEBMI	URBMI	NCMS
Inception year	1998	2007	2003
Eligible population	Urban employees in formal sectors	Urban residents without formal jobs or who are unemployed: children, students, elderly people	Rural residents
Participation	Mandatory	Voluntary	Voluntary
Enrolment	274.2 million (2013)	299.1 million (2013)	0.80 billion (2013)
Administration	Chinese Ministry of Human Resources and Social Security	Chinese Ministry of Human Resources and Social Security	Chinese National Health Commission
Risk-pooling unit	Municipal level	Municipal level	County level
Source of funding	Contributory (8% of annual payroll: 6% from employers and 2% from employees)	Government subsidies (70%) and individual premiums (30%)	Government subsidies (80%) and individual premiums (20%)
Per capita fund (2013)	US$424.70	US$66.20	US$61.20
Service coverage	Inpatient	Inpatient, catastrophic illness	Inpatient, outpatient

References: Qiu. et al. [8], National Bureau of Statistics [9], Su et al. [10]

Since China’s reform and opening to the outside world at the end of the 1970s, with the development of industrialization, urbanization, and economic growth, a large number of rural-to-urban migrant workers in China have entered cities for employment opportunities [19]. The number of rural-to-urban migrant workers has been increasing dramatically, from approximately 2 million in 1983 to 147 million in 2005 and to 281 million in 2016 [19, 20]. Although rural-to-urban migrant workers are widely considered the transformational force driving economic growth in China [21, 22], their health status and healthcare utilization have not received enough attention.

To increase the overall health insurance coverage of rural-to-urban migrant workers, through continuous reform, migrant workers have been entitled to participate in one or more insurance schemes. Migrant workers who have formal jobs can enrol in UEBMI, while other migrant workers without formal jobs but who have a hukou in their host city can choose to participate in the URBMI of their local city or the NRCMS of their registered place of residence. Although migrant workers are officially covered by basic health insurance, due to its “urban-rural duality” characteristics, the health insurance system still has different effects on the medical service utilization and health status of migrant workers who participate in different urban and rural health insurance schemes. On the one hand, the urban and rural health insurance systems of China are fragmented, with no transfers between the risk pools despite the differential health care needs and revenue bases [14]. Compared with migrant workers who participate in the NRCMS, migrant workers who participate in urban health insurance can enjoy better medical services and acquire a higher reimbursement level. This implies that migrant workers who participate in urban health insurance and the NRCMS have varying degrees of access to health care, which may influence their health care utilization and which may have different effects on their health status. On the other hand, the urban and rural health insurance schemes have different management requirements. The NRCMS requires migrant workers to participate in insurance and reimburse medical expenses at their registered place of residence. Such a policy requirement of binding health insurance to the household registration system will increase the burden on migrant workers, as their benefits and reimbursements are generally not portable across counties and provinces; additionally, it is normally impractical for migrant workers to return home to where their hukou is situated for healthcare and other social benefits while working in an urban host city [21]. This situation may lead migrant workers using the NRCMS to put off seeing a doctor, which may ultimately affect their self-rated health (SRH). However, migrant workers are also facing many restrictions on participation in urban health insurance. UEBMI, which is paid jointly by enterprises and employees, requires insured individuals to have a job with a stable or formal contract. However, many migrant workers can only engage in temporary, labour-intensive and less skilled jobs with long working hours and lower pay in part due to their lower education or lack of skills training [23]. Disadvantaged migrant workers with unstable and informal jobs are easily excluded from UEBMI. Additionally, with regard to URBMI, because part of the URBMI premium comes from the municipal government, in general, migrant workers are ineligible for the local URBMI programmes in their host cities unless they obtain a hukou for their host city [21]. At the same time, migrant workers who participate in urban health insurance, including UEBMI and URBMI, may face higher medical expenses when they seek health care in urban hospitals, which will increase their economic burden and affect their SRH status. Such differences in the urban and rural health insurance systems may have different effects on the SRH status of migrant workers.

Previous studies have suggested that in other nations, differences in health insurance play an important role in creating health disparities [24–27]. For example, Brook et al. [27] found that a medical insurance scheme with higher compensation will lead residents to use more medical resources. Controlling for income level and initial health status, they found that completely free medical insurance can significantly improve the condition of persons with poor vision and low-income persons with high blood pressure; however, it had no significant effects on eight other measures of health status and healthy habits [28]. Compared with studies in other countries, less attention has been paid to the impact of China’s different health insurance schemes on health, especially on the health of migrant workers. Instead, some studies have analysed individual health insurance plans, focusing more on elderly individuals and rural residents [28–31]. Although Qin et al. [17] compared three health insurance schemes and assessed whether participating in health insurance programs promoted migrant workers’ use of medical services and improved their SRH, the study was based on an early-period database, and the long-term effects of some insurance programmes – such as URBMI and the NRCMS – were not fully revealed due to their relatively short implementation time. One concern is that under the different health insurance systems in China’s urban and rural areas, migrant workers may vary their health-seeking behaviours, leading to disparities in health status. To date, there is no existing study that compares the different effects of urban and rural health insurance on the health of migrant workers in China. Given this background, this study seeks to compare urban and rural health insurance from the perspective of urban and rural segmentation in China, examining whether the urban and rural health insurance schemes have created disparities in the SRH of migrant workers and improved their health, to provide a reference for future research and to promote social equity.

Notably, the seemingly positive link between insurance coverage and health may not indicate a causal relationship [17] because insurance participation and health status can be simultaneously affected by unobserved factors, such as individuals’ cultural background and risk preferences [32]. In turn, these factors lead to endogeneity problems with regard to insurance participation and may cause estimation bias in the impacts of such participations [33]. To address this issue, various identification methods are used to evaluate the impact of health insurance on the health status of different groups of people. For example, Ludema et al. [34] used Cox proportional hazards models to estimate the effect of the AIDS Drug Assistance Program (ADAP) on the health outcomes of HIV-infected women in the United States, finding that the ADAP contributed to viral load suppression. Finkelstein and McKnight [35] used data from the 1960s and the difference-in-differences estimation method to find that in the first 10 years, the establishment of universal health insurance for the elderly population had no discernible impact on their mortality. Card et al. [36] used a regression discontinuity approach to estimate the impact of Medicare coverage on mortality and noted some improvements in SRH at age 65. Other studies used instrumental variable (IV) estimation to resolve the problem of endogeneity [37, 38]. Dor et al. [37] used the state-level unemployment rate, unionization, and tax rates as instrumental variables to estimate the effects of private insurance on the health of near-elderly individuals. Hadley and Whiteman [38] used spouses’ prior union status, immigrant status, years in the United States and involuntary job loss in the past 5 years as instrumental variables to address the endogeneity of insurance and found that continuous private health insurance significantly improves the health of near-elderly individuals.

## Methods

### Setting and participants

In this study, data were obtained from a cross-sectional survey conducted from June to December 2016 in Chengdu, the capital of Sichuan Province, and Kunming, the capital of Yunnan Province, two large cities in Southwest China that have attracted many migrant workers from rural areas. In 2016, Chengdu had a population of 15.9 million and a migrant population of approximately 6.3 million [39], and Kunming had a permanent population of 6.7 million [40]. A multistage stratified cluster sampling method was used to acquire the sample. First, based on social and economic conditions, we selected two central districts in Chengdu and one district surrounding Chengdu; additionally, we selected one central district in Kunming and one district surrounding Kunming. Next, 1–2 communities were randomly selected in each district. Then, based on the characteristics of the community population, each community was allocated into 2–5 residential areas. Finally, based on the number of inhabitants in each residential area, 2 buildings were selected from each residential area, and face-to-face household surveys were conducted with all eligible residents in the selected buildings.

The population under consideration in the paper consists of rural-to-urban migrant workers in the sample cities. Due to the high floating nature of this group (migrant workers change their places of residence frequently), if we use longitudinal research, there will be significant sample attrition across years, resulting in a highly unbalanced panel. The difficulty in recovering the information on missing observations rules out the possibility of conducting longitudinal studies on the migrant worker population. Therefore, we use 2016 survey data. Another advantage of the cross-sectional approach is the better utilization of the new information brought by the observations.

In this article, the migrant worker sample was selected under the following criteria: (1) having a rural hukou; (2) engaging in non-agricultural work in an urban area; and (3) being over the age of 18. After eliminating missing data, 8507 eligible participants were included in our final analysis.

### Data collection

The study sample was based on the family unit, and each family chose the one member most familiar with the family’s situation as the respondent. On the basis of informed consent, each participant participated in a face-to-face interview using a questionnaire administered by trained investigators, and the questionnaire was completed anonymously. The content of the questionnaire included the respondent’s physical and mental health status, health-related behaviours, demands for health services, medical insurance situation, and socio-demographic information such as gender, age, income status, and work situation. Data were collected by trained master’s or doctoral students from the West China School of Public Health, Sichuan University, Chengdu, China.

### Variables

We use the response to the following question related to SRH as our outcome of interest: “How would you rate your health at the present time?” The response options were “very good,” “good,” “moderate,” “bad,” and “very bad.” As an individual perception of health, SRH has been shown to be important, independent of the objective presence of any disease [41], and it has been widely used in population-based surveys and research [42, 43]. We rank the responses on a five-level scale as follows: 1 = very bad, 2 = bad, 3 = moderate, 4 = good, and 5 = very good.

The independent variables consist of two main health insurance variables: urban health insurance (UEBMI plus URBMI) and rural health insurance (NRCMS). Each adult in the study was asked whether he or she had health insurance at the survey time. If the answer was yes, he or she was asked whether the health insurance plan was the new rural cooperative medical system, urban employee basic medical insurance, urban resident basic medical insurance, or non-social insurance. Non-social insurance includes other health insurance plans (such as commercial health insurance). Some respondents with other health insurance or under “unknown” answers were excluded from this study due to small sample sizes. The problem with health insurance in the questionnaire is that the participant is required to choose only one major option; at the same time, we merge UEBMI and URBMI into urban health insurance. Thus, in our analysis, each respondent has only one choice of health insurance, that is, one kind of urban insurance, one kind of rural insurance or uninsured.

Based on the previous literature, other control variables include demographic characteristics, occupational characteristics, objective health indicators, and health-related behaviours: gender, age, marital status, education, monthly income, years living in this city, occupation, employment position, smoking, alcohol consumption, being chronic disease patients, and being sick during the past 2 weeks.

To determine smoking status, the question asked was “Have you ever smoked cigarettes?” If the participants had been diagnosed by doctors as suffering from any chronic disease, including hypertension, diabetes, myocardial infarction, stroke, cancer, and other chronic diseases, we identified them as chronic disease patients. We judge whether the participants have been sick during the past 2 weeks by whether they have suffered from any chronic or acute disease during the past 2 weeks.

Two variables, income source and the number of family members who have lived in the household during the past 6 months, are adopted as instrumental variables. Income source was measured using the following question: “From where do you receive most of your income?” Five response options were provided as follows: (1) work, (2) subsidies from parents, (3) sponsorship from relatives and friends, (4) the government poverty allowance, and (5) other sources. The answers were ranked at five levels based on the varying degrees of self-initiative and income acquisition stability. Self-initiative refers to the extent to which a person can determine his or her own income. “Gaining income from work” is the strongest in terms of self-initiative and the most stable in terms of income acquisition. Conversely, “other sources” is the weakest in terms of self-initiative and the most unstable in terms of income acquisition. Therefore, the variable definition of income source is 1 = gaining income from work and 5 = other sources. The number of family members who have lived in the household during the past 6 months is measured using the following question: “During the past six months, how many members in your household have lived at home?”

Effective instrumental variables are needed to satisfy two conditions: first, instrumental variables are correlated with endogenous explanatory variables; second, instrumental variables are uncorrelated with unobservable errors. We use two variables, income source and the number of family members who have lived in the household during the past 6 months, as instrumental variables. The income source is highly correlated with migrant workers’ insurance enrolment. Different types of income sources determine whether migrant workers choose to participate in health insurance and in what kind of health insurance schemes they participate. The stronger the individual self-initiative and the more stable the expected income, the higher the possibility that migrant workers will participate in urban health insurance schemes that are more expensive but that provide a higher level of security. However, different types of personal income sources have no direct impact on SRH. The number of family members who have lived in the household during the past 6 months is also correlated with migrant workers’ insurance participation. Because the NRCMS is a kind of voluntary health insurance with the household (or family) as the insured unit, either all household members or no household members participate in the NRCMS plan [26]. Therefore, if the number of family members who have lived in the household during the past 6 months is larger, more members have lived at home. Considering that most family members stay in their hometown, migrant workers will be more likely to participate in the NRCMS in rural areas. On the other hand, the number of family members who have lived in the household during the past 6 months has little or no direct relationship with migrant workers’ SRH.

### Statistical analysis

We employ bivariate and multivariate methods in the empirical analyses. We compare the SRH and other control variables of migrant workers with different urban and rural health insurance plans by conducting a chi-square test. In this study, SRH is used as a continuous measure. Thus, ordinary least squares (OLS) and instrumental variable (IV) methods are employed to study the relationship between urban and rural health insurance and the SRH of migrant workers. Our estimation models are as follows:

### Ordinary least squares (OLS) model

1 $$ Y={\beta}_1 UBINSU+{\beta}_2 RUINSU+{\beta}_3X+\omega $$

where *Y* is migrant workers’ SRH, *UBINSU* indicates whether migrant workers participate in urban health insurance (UEBMI or URBMI), *RUINSU* indicates whether migrant workers participate in rural health insurance (NRCMS), and *X* is a set of control variables, including gender, age, educational level, income, etc.

### Instrumental variable (IV) approach

The OLS model assumes that health insurance is exogenous, which means that health insurance can affect health without being influenced by other factors. However, as mentioned above, deciding to participate in health insurance is endogenous to health, which means that health insurance may be related to some unobserved factors. In brief, some unobservable factors may influence the decision to purchase health insurance, which also influences health status [21]. If we do not consider this and do nothing to address the endogeneity problem, we may obtain inconsistent and biased estimates of the relationship between health insurance and SRH. To tackle this problem, on the basis of multivariate regression, we further use the following two-stage instrumental variable approach to adjust for possible bias in our model:

2 $$ Y={\beta}_1 UBINSU+{\beta}_2\mathrm{R} UINSU+{\beta}_3X+{\omega}_{1\kern2em }\kern5em $$

3 $$ RUINSU={\gamma}_1Z+{\gamma}_2X+{\omega}_4\kern13em $$

4 $$ UBINSU={\alpha}_1Z+{\alpha}_2X+{\omega}_{2\kern2em }\kern11.25em $$

Equation (2) is the relationship between urban and rural health insurance and the SRH of migrant workers. Equation (3) and Eq. (4) are equations of migrant workers decision-making with regard to participating in rural and urban health insurance, respectively. Z is the instrumental variables, and X is the set of control variables, including gender, age, educational level, income, etc.

## Results

Table 2 reports the descriptive statistics for the dependent and independent variables. Approximately 25% of the respondents are covered by urban health insurance (including UEBMI and URBMI), 60% are covered by the NRCMS, and 15% are uninsured. Overall, migrant workers participating in urban health insurance are healthier, with approximately 40% of them having good and very good SRH, while 22% of NRCMS members and 23% of uninsured respondents evaluate their own health status as good and very good (*p* < 0.0001). Regarding the other control variables, we find significant differences in terms of age, marital status, education, monthly income, years living in the city, occupation, employment position, and smoking (*p* < 0.0001). Compared with migrant workers enrolled in the NRCMS and without insurance, migrant workers enrolled in urban health insurance are younger, have a better educational level and occupation, and have more stable employment. However, no statistically significant differences were found in terms of gender, alcohol consumption, being chronic disease patients, or being sick during the past 2 weeks (*p* > 0.05).

**Table 2:** Descriptive statistics

Variable	Urban health insurance (***N*** = 2111)	NRCMS^**a**^ (***N*** = 5123)	Uninsured (***N*** = 1273)	***p***
**Self-rated health (%)**				< 0.0001
Very bad	3.51	4.55	4.71	
Bad	28.47	50.11	50.59	
Moderate	27.66	23.01	21.52	
Good	32.64	18.49	19.01	
Very good	7.72	3.85	4.16	
**Health insurance coverage**				< 0.0001
**Insured by urban health insurance (%)**				
Yes	100.00	0.00	0.00	
No	0.00	100.00	0.00	
**Insured by the NRCMS (%)**				
Yes	0.00	100.00	0.00	
No	100.00	0.00	0.00	
**Insured by neither urban health insurance nor the NRCMS (%)**				
Yes	0.00	0.00	100.00	
No	0.00	0.00	0.00	
**Gender (%)**				0.372
Male	52.87	52.49	50.51	
Female	47.13	47.51	49.49	
**Age (mean)**	35.22	40.71	40.90	< 0.0001
**Age (years) (%)**				< 0.0001
18–29	49.36	25.18	31.19	
30–45	30.93	41.17	32.76	
46–59	10.75	21.57	21.05	
60 +	8.95	12.08	15.00	
**Marital status (%)**				< 0.0001
Having a spouse	58.55	80.58	70.54	
Single	41.45	19.42	29.46	
**Education (%)**				< 0.0001
Primary school and below	15.73	34.73	36.29	
Lower high school	31.69	39.08	31.89	
Upper high school	33.11	19.95	19.95	
University and above	19.47	6.25	11.86	
**Monthly income (yuan) (%)**				< 0.0001
≤ 2000	17.86	31.11	39.91	
2000-3000	48.18	33.85	30.16	
3001-5000	25.34	26.04	21.45	
≥ 5000	8.62	9.00	8.48	
**Years living in this city (years) (%)**				< 0.0001
≤ 1	12.03	46.91	46.19	
1–5	30.41	10.83	13.83	
6–10	29.42	6.42	10.84	
≥ 11	28.14	35.84	29.14	
**Occupation (%)**				< 0.0001
Professional/administrator	24.92	6.40	10.37	
Worker	54.19	31.90	29.77	
Service worker	20.89	61.70	59.86	
**Employment position (%)**				
Permanent employee	17.05	12.90	8.80	< 0.0001
Contractor	58.93	22.53	24.43	
Temporary worker	24.02	64.57	66.77	
**Smoker (%)**				< 0.0001
Yes	8.34	22.12	18.30	
No	91.66	77.88	81.70	
**Alcohol consumption (%)**				0.475
Yes	18.95	17.96	19.09	
No	81.05	82.04	80.91	
**Chronic disease patient (%)**				0.084
Yes	6.77	8.32	8.09	
No	93.23	91.68	91.91	
**Sick during past the 2 weeks (%)**				0.495
Yes	11.13	11.09	9.98	
No	88.87	88.91	90.02	
**Total**	**100.00**	**100.00**	**100.00**	

^**a**^NRCMS represents rural health insurance

Table 3 shows the composition of the income sources of migrant workers, grouped by urban health insurance and the NRCMS. Most of the income of migrant workers with both urban and rural health insurance is obtained from employment, which accounts for 90% of the income of migrant workers enrolled in urban health insurance and 89% of the income of migrant workers enrolled in the NRCMS, followed by other sources for both migrant worker groups, accounting for 6–7% (*p* = 0.018).

**Table 3:** Income sources of migrant workers, grouped by urban and rural health insurance

Variable	Urban Health Insurance (***N*** = 2052)	NRCMS (***N*** = 4601)	***p***
**Income source (%)**			0.018
Work	90.06	89.18	
Parents	0.49	0.35	
Sponsorship from relatives and friends	0.88	1.91	
Government poverty allowance	1.46	1.85	
Other sources	7.11	6.71	
**Total**	**100.00**	**100.00**	

Table 4 shows the composition of the number of family members who have lived in the household during the past 6 months, grouped by urban health insurance and the NRCMS. During the past 6 months, 80% of migrant workers with both urban and rural insurance had no family members living at home where their household registration is located, followed by 5.02% of migrant workers with urban health insurance. Additionally, 6.40% of migrant workers participating in the NRCMS had 3 family members living at home (*p* = 0.05).

**Table 4:** Number of family members, grouped by urban and rural health insurance

	Urban Health Insurance (***N*** = 2111)	NRCMS (***N*** = 5123)	***p***
**Number of family members who have lived in the household during the past 6 months (%)**			0.05
0	80.25	80.05	
1	1.18	1.29	
2	4.50	3.24	
3	5.02	6.40	
4	4.12	4.06	
5	4.45	4.18	
6	0.43	0.74	
7	0.05	0.04	
**Total**	**100.00**	**100.00**	

Table 5 shows the estimated results of using the OLS regression model to examine the association between urban and rural health insurance and the respondents’ SRH. The OLS regression model is used to assume that health insurance is an exogenous variable; that is, being insured is not affected by individuals’ health, and there are no unobservable factors that jointly affect insurance and health. The data in Table 5 show that the SRH of migrant workers participating in the NRCMS is not significantly different from the SRH of migrant workers without insurance. Migrant workers with urban health insurance have better SRH than those without insurance, with a coefficient of 0.206 (95% CI, 0.134, 0.279), which shows that migrant workers with urban health insurance rate their health 0.206 higher than those without health insurance.

**Table 5:** OLS regression estimates of migrant workers’ SRH

	Model (1)	
	Coeff.	(95% CI)
**Health insurance coverage No insurance (reference)**		
NRCMS	0.002	(−0.058, 0.063)
Urban health insurance	0.206***	(0.134, 0.279)
Observations	8507	
*R*^*2*^	0.144	

Note: R^2^ is the coefficient of determinant. *p < 0.10; **p < 0.05; ***p < 0.01. The estimation model controlled the variables of gender, age, marital status, education, years living in the city, monthly income, occupation, employment position, being chronic disease patients, being sick during the past 2 weeks, smoking, and alcohol consumption

The above OLS model implicitly assumes that health insurance participation is exogenous. However, as we pointed out earlier, different health outcomes for insured and uninsured individuals may be the result of differences in health insurance or other differences caused by some observed or unobserved factors, making health insurance endogenous. Because of the potential for confounding unobservable factors, the OLS model may lead to deviation of the estimation results. Therefore, we further use IV estimation to adjust for possible biases

We first test whether the two instrumental variables, income source and the number of family members who have lived in the household during the past 6 months, are appropriate before we use the IV method to analyse the health effects of urban and rural health insurance. The first test is the weak instrumental variable test, which is used to test whether these two instrumental variables are correlated with the suspected endogenous variables. First, from the regressions of the two first-stage insurance status equations of models (2) and (3) in Table 6, we find that the two instrumental variables, income source and the number of family members who have lived in the household during the past 6 months, are statistically significant at the 1% level, indicating that the two instrumental variables have an impact on the suspected endogenous variables. Second, according to the rule of thumb proposed by Stock et al. [44], the F-statistic of the first-stage regression is 10, which is a critical point of experience. If the F-statistic is greater than 10, the original hypothesis that there is a weak instrumental variable can be rejected, and there will be no need to worry about problems caused by a weak instrumental variable [44]. From the test results in Table 6, the F-statistics of the first-stage regression of the two models far exceed the critical value standard of F = 10. The F-statistics of the health status statistical model under urban health insurance and the NRCMS are 11.638 and 39.946, respectively. This shows that both instrumental variables pass the weak instrumental variable test

**Table 6:** Two-stage IV multivariate regression estimations of immigrant workers’ health

X	First-stage regression NRCMS (2)	First-stage regression Urban Health Insurance (3)	SRH (4)
	Coeff. (95% CI)	Coeff. (95% CI)	Coeff. (95% CI)
**Health insurance coverage No insurance (reference)**			
NRCMS			−0.419 (−1.226, 0.389)
Urban health insurance			2.753^**^ (0.504,5.002)
Income source	−0.030^***^ (− 0.049, − 0.011)	0.015^***^ (0.004, 0.026)	
Number of family members who have lived in the household during the past 6 months	−0.051^***^ (− 0.065, − 0.038)	−0.019^***^ (− 0.028, − 0.010)	
Contact	0.741^***^ (0.683, 0.801)	0.082^***^ (0.037, 0.127)	
Observations	7696	7696	7696
Weak instrumental variable tests			
F-statistics	39.946	11.638	

Notes: *p < 0.10; **p < 0.05; ***p < 0.01. The estimation model controlled the variables of gender, age, marital status, education, years living in this city, monthly income, occupation, employment position, being chronic disease patients, being sick during the past 2 weeks, smoking, and alcohol consumption

Second, we test the exogeneity of the instrumental variables, which means that the instrumental variables should not directly correlate with unobservable errors in the insurance models. Although it is theoretically impossible to prove that an instrumental variable is uncorrelated with the outcome measure, based on prior studies [17, 45, 46], we use an indirect testing method by applying an OLS model to estimate the correlation between the two instrumental variables and migrant workers’ SRH. The model includes two variables: income source and the number of family members who have lived in the household during the past 6 months. Additional file 1: Table S1 reports the regression results and shows that our instrumental variables are not statistically significant, suggesting that such a direct correlation in our sample is not significant

Table 6 presents the estimation results of the two-stage IV multivariate regression of migrant workers’ SRH. The IV model in Table 6 shows that, similar to the OLS model, the SRH of migrant workers enrolled in the NRCMS is not significantly different from the SRH of migrant workers without insurance, while migrant workers with urban health insurance have better SRH than those without insurance. However, there is a difference from the OLS results. That is, in model (4), the coefficient of urban health insurance is 2.753 (95% CI: 0.504, 5.002), which indicates that the health status of migrant workers can be improved by 2.753 units by participating in urban health insurance, which is much higher than the OLS estimate of 0.206. This result indicates that after controlling the endogenous problems of insurance, migrant workers with urban health insurance are more likely to report better health than those without urban health insurance.

Table 7 shows the results of testing the relationship between urban and rural health insurance and the SRH of migrant workers by gender. We still use income source and the number of family members who have lived in the household during the past 6 months as instrumental variables. The corresponding test results show that the models are endogenous and that the instrumental variables are effective. From the regression results of Table 7, the results of the test grouped by gender are basically consistent with the previous analysis results. The SRH of migrant workers enrolled in the NRCMS is not significantly different from the SRH of migrant workers without insurance. Based on the coefficient estimates, urban health insurance has a potentially positive influence on male and female migrant workers’ SRH, but only the female model demonstrates a statistically significant impact: compared with uninsured migrant workers, female migrant workers enrolled in urban health insurance have an increased probability of reporting better health. More specifically, we find differences between the sexes in the impact of urban health insurance and the NRCMS on the health of migrant workers. Urban health insurance and rural health insurance have a greater impact on women’s health than on men’s health.

**Table 7:** IV estimation results of the health of immigrant workers, grouped by gender

	Model	
Variables	Male (5)	Female (6)
	Coeff. (95% CI)	Coeff. (95% CI)
**Health insurance coverage** **No insurance (reference)**		
NRCMS	−0.054(−0.927, 0.819)	−0. 528 (−2.078, 1.022)
Urban health insurance	0.664(−1.609, 2.938)	5.483*(−0.646, 11.613)
Contact	2.540***(2.136, 2.944)	3.270*** (2.085, 4.454)
Number	4201	3495

Notes: *p < 0.10; **p < 0.05; ***p < 0.01. The estimates shown in Table 7 are the second-stage regression results, and the endogeneity of insurance participation was controlled using instrumental variables. The estimation model controlled the variables of age, marital status, education, years living in this city, monthly income, occupation, employment position, being chronic disease patients, being sick during the past 2 weeks, smoking, and alcohol consumption

## Discussion

Based on survey data on Southwest China, this study uses the IV regression method, in which income source and the number of family members who have lived in the household during the past 6 months, which are not directly related to health, are chosen as instrumental variables, for further estimation. We find substantial disparities in migrant workers’ health status based on the different types of urban and rural health insurance. We find that the SRH of migrant workers enrolled in the NRCMS is not significantly different from the SRH of migrant workers without insurance, while urban health insurance is associated with significant improvements in the health of migrant workers after controlling for the endogeneity problems of health insurance. Overall, compared with the NRCMS, migrant workers participating in urban health insurance, including UEBMI and URBMI, were more likely to have better SRH.

The NRCMS is shown to be negatively correlated with the SRH of migrant workers, although there is no statistically significant correlation. Much of the reason for this result may be the institutional barriers in the NRCMS platform, which hinder rural-to-urban migrant workers from making full use of the benefits of the insurance. The NRCMS is typically the first choice of many migrant workers due to its low premium and high government subsidy. However, the NRCMS requires its enrolees to seek medical care in their registered county of residence (hukou location), and its policies discriminate against out-of-county medical utilization, such as physician referral requirements and lower reimbursement rates [9]. For migrant workers, this geographic limitation means a higher opportunity cost of seeking medical care in cities [9]. However, it is also normally impractical for migrant workers to return home to where their hukou is situated for health care benefits while working in an urban host city because doing so will require them to spend considerable time and money to return to the countryside to see a doctor. Therefore, NRCMS policies limit the actual protection of migrant enrolees who rely on urban medical resources. Migrant workers may vary their health-seeking behaviours due to the institutional barriers associated with the NRCMS. Because of the difficulties in being reimbursed for returning to the country or the inability to afford the high medical expenses in the city [47], they often choose self-medication or not to see a doctor when they are ill even if they have participated in the NRCMS [48]. Their unhealthy health-seeking behaviour may result in grave health consequences in the long run [47].

Earlier research conducted by Qin et al. [17] found that UEBMI had a positive influence on migrant workers’ SRH. Similar to their study, our study goes further and finds that urban health insurance (including UEBMI and URBMI) can improve the SRH status of migrant workers. One possible explanation is that the relatively well-established system of urban health insurance is conducive for migrant worker enrolees to benefit from their coverage. Compared with rural health insurance, urban health insurance is significantly more generous [26], with a higher reimbursement percentage. This disparity in the reimbursement percentage may reflect more comprehensive benefit packages in urban health insurance, in contrast to the high deductibles/co-payments and low maximum reimbursement caps that part of the NRCMS [26]. The NRCMS scheme generally does not reimburse expensive outpatient services for chronic conditions; indeed, in its early years, the reimbursement rate was as low as 11–15% [26]. At the same time, compared with the NRCMS, urban health insurance has a better reimbursement system; thus, migrant workers with urban health insurance are more likely to receive preventive health services [26, 48]. On the other hand, migrant workers who participate in urban health insurance can see a doctor more conveniently because they are in a city.

Based on the robustness test of the relationship between urban and rural basic health insurance on the SRH of migrant workers, we find gender differences in the health impact of urban health insurance and the NRCMS on migrant workers. There is no significant correlation between the NRCMS and migrant workers’ SRH in either the male or female groups. Urban health insurance is associated with considerable improvement in migrant female workers’ evaluations of their own health.

The results from the present study should be interpreted with caution, as there are some limitations. First, although we use the OLS model and IV estimation to control the endogeneity problem of health insurance as much as possible, our survey takes the form of a cross-sectional survey rather than a longitudinal survey. We are therefore unable to control for unobserved individual characteristics that are consistent over time (e.g., genetic factors and family backgrounds). The failure to control these factors may have led to bias in our estimation of the correlation between health insurance and SRH. Second, although it is a strong predictor of health status, SRH is still a subjective measure. All data in our study relied on the self-report of the respondents. As with any other questionnaire study, the subjects’ answers may be affected by various kinds of bias, such as social desirability bias. Third, this study is part of a larger study; therefore, the research questions can only be understood within the boundaries of the original study. Some indicators were not integrated in our study due to the original focus of the study. Finally, in our study, the survey data came from only two cities in Southwest China and may not accurately represent the whole country.

## Conclusions

Health insurance is an important component of a nation’s social security system and is considered an essential tool for ensuring equal access to medical resources and promoting population health [7]. As a combination of objective health and subjective mental health, SRH is an individual’s comprehensive evaluation of his or her whole body and mental state [28]. Therefore, exploring the correlation between urban and rural health insurance and the SRH of immigrant workers in China holds significance. This study has demonstrated that disparities exist in the relationship between urban and rural health insurance and the SRH status of migrant workers. Compared with the NRCMS, urban health insurance has a more positive correlation with the SRH of migrant workers.

A more generous health insurance scheme is generally associated with improved population heath and can reduce health inequities, particularly for vulnerable groups. Despite China’s continuous reform efforts to provide migrant workers with more opportunities to participate in urban health insurance schemes, the inequity in the distribution of basic health insurance remains [21]. Urban health insurance still maintains restrictions on migrant workers. Due to their lower education, unstable employment, lack of long-term contracts and lack of local household registration in host cities, many migrant workers are still excluded from the urban health insurance system. However, compared with the NRCMS, urban health insurance, including UEBMI and URBMI, has a more generous benefit package. Meanwhile, migrant workers spend more time in cities than in rural areas over the course of a whole year because they need to work in urban cities. Therefore, relaxing restrictions on urban basic health insurance and encouraging migrant workers to participate in the urban health insurance system can not only enable such workers to obtain a higher medical expense reimbursement rate but also improve the accessibility of medical services and the health status of migrant workers.

Although there are more health insurance schemes for migrant workers to choose from, the NRCMS is still the first choice for most migrant workers. However, some discriminatory policies in the NRCMS, such as acquiring lower reimbursement rates if enrolees see an out-of-county doctor, have created obstacles to migrant workers’ utilization of medical services, which is negatively correlated with the SRH status of migrant workers. Therefore, policy makers should gradually eliminate the discriminatory policies of the NRCMS and promote the role of the NRCMS in improving the accessibility of medical services for migrant workers.

In addition, the fragmentation of China’s whole health insurance system not only violates the principle of fairness but also is contrary to the characteristics of the high mobility of migrant workers, which is not conducive to economic and social development. Thus, expanding the coverage of the current schemes alone is unlikely to be effective in satisfying migrant workers’ medical needs, as urban and rural health insurance schemes are administered independently. Reducing this fragmentation is necessary to promote equity in acquiring health services [5]. Thus, policy makers should fill the “gaps” between platforms and continue to promote the integration of urban and rural health insurance. Additionally, they should make insurance benefits portable and transferable across employment sectors and geographic regions.

## Supplementary Information

**Additional file 1: Table S1.**

## Data Availability

The datasets used and/or analysed during the current study are available from the corresponding author on reasonable request.

## Abbreviations

UEBMI: Urban employee basic medical insurance
URBMI: Urban resident basic medical insurance
NRCMS: New rural cooperative medical system
SRH: Self-rated health
IV: Instrumental variable
ADAP: AIDS Drug Assistance Program
OLS: Ordinary least squares
