# Supplementary material for: The relationship between urban and rural health insurance and the self-rated health of migrant workers in Southwest China
Source: BMC Health Serv Res. 2021 Jun 29;21:614. doi: 10.1186/s12913-021-06646-3 (PMC8240306; doi:10.1186/s12913-021-06646-3)
Supplement: Supplementary file 1 — Additional file 1: Table S1. [file 12913_2021_6646_MOESM1_ESM.docx]

**Appendix Table A**

**Instrumental variables and migrant workers’ SRH—Regression results**

| **SRH** | **Coeff. (95% CI)** | |
| --- | --- | --- |
| Income source | -0.039 | (-0.110, 0.031) |
| Number of family members who have lived at home during the past six months | 0.0086 | (-0.006, 0.022) |
| **Gender (Female)** |  |  |
| Male | -0.130 *** | (-0.177, -0.084) |
| **Age (18–29)** |  |  |
| 30-45 | -0.075 ** | (-0.133, -0.017) |
| 46-59 | 0.012 | (-0.059, 0.084) |
| 60 + | -0.021 | (-0.114, 0.072) |
| **Marital status (Single)** |  |  |
| Having a spouse | -0.137 *** | (-0.193, -0.082) |
| **Education (Primary school and below)** |  |  |
| Lower high school | -0.012 | (-0.065, 0.041) |
| Upper high school | -0.060 * | (-0.124, 0.004) |
| University and above | -0.181 *** | (-0.271, -0.090) |
| **Monthly income (≤2,000)** |  |  |
| 2,000–3,000 | 0.048 | (-0.010, 0.107) |
| 3,001–5,000 | 0.097 *** | (0.024, 0.150) |
| ≥5,000 | 0.201 *** | (0.112, 0.290) |
| Years living in this city | -0.016* | （-0.032, 0.0001） |
| **Occupation (Service worker)** |  |  |
| Worker | 0.387 *** | (0.331, 0.442) |
| Professional/administrator | 0.437 *** | (0.353, 0.522) |
| **Employment position (Temporary worker)** |  |  |
| Permanent employee | 0.136*** | (0.065, 0.208) |
| Contractor | 0.227*** | (0.166, 0.287) |
| Smoker (Yes=1) | -0.167*** | (-0.225, -0.110) |
| Alcohol consumption (Yes=1) | 0.094*** | (0.038, 0.150) |
| Chronic disease patient (Yes=1) | -0.373*** | (-0.474, -0.272) |
| Sick during the past 2 weeks (Yes=1) | -0.218*** | (-0.304, -0.131) |
| Observations | 8507 | |
| *R^2^* | 0.928 | |

**Note**: R^2^ is the coefficient of determination. **p* < 0.10; ***p* < 0.05; ****p* < 0.01.
